# Glances at the Hospitals

**Published:** 1905-12-30

**Authors:** 


### GLANCES AT THE HOSPITALS.

### THE CANCER HOSPITAL, BROMPTON, S.W.

The ordinary income amounted to £8,195, and the ordinary expenditure to £12,367. Legacies to the amount of £14,888 were received, and a sum of £4,499 was transferred from the capital account to cover the excess of expenditure over income. The annual subscriptions, the donations, and the dividends received all show a slight increase over those of the previous year, but the subscriptions and donations must be greatly increased if the hospital is to pay its way without drawing on its capital account. London sent 507 of the in-patients admitted during the year, and 204 were admitted from 27 English counties. The total number of new patients was 2,425, and of these 711 were in-patients, the average daily number resident being 84. On looking at the table showing the parts affected, among the in-patients we notice that the disease was in the breast in 152 cases; in the uterus in 124; in the ovary 21; in the lip, mouth, tongue, œsophagus, or larynx in 60. Of these 60 instances 51 were men and 9 women, showing how much more frequent the disease is in these organs in the male sex than it is in the female; but in the rectum out of 48 cases 21 were in men and 27 in women. Of the year's new cases 501 were treated by operation and 210 without operative interference. Of the total, 621 were discharged convalescent or relieved, and 108 died. An anonymous donor generously placed a quantity of radium at the disposal of the surgeons, and Dr. Snow went to Vienna to see the cases treated by radium under Professor Exner. Since then the treatment has been commenced at Brompton, but had not been completed at the date of the report. We should have been glad of somewhat fuller details in some cases; and we miss the anæsthetic table.

### GREAT YARMOUTH HOSPITAL.

The total ordinary income was £2,577, and the total ordinary expenditure was £2,515, which shows a small balance on the right side, and as the Committee remark, the income just meets the expenditure. This is a financial state that a great many hospitals must envy; but, very properly, the Yarmouth Committee want a larger margin, and they hope that "additional subscriptions may be forthcoming." On looking over the subscription list we notice that the Fishing Industry Collection only amounted to £49, and although this sum might be increased by additions of various small amounts from workmen's societies, etc., it is clear that the total falls a long way short of what it ought to be in a town of 50,000 inhabitants. We are glad to note that Mr. William Arnold presented a complete *x*-ray apparatus to the hospital. The hospital contains 60 beds, and the average number occupied was 43. The average of each patient's stay in the wards was 35 days, which is certainly higher than the average of similar institutions. The total number of in-patients was 445, and of these 374 were cured or relieved, 37 died, and 34 remained under treatment. The average cost per bed occupied was £49 3s. The statistical tables are arranged so as to show the total numbers under treatment for various diseases, the sexes, the numbers cured, relieved, died, and remaining in the hospital. There were 7 cases of pneumonia with no death, and 20 cases of acute rheumatism without a death. Altogether 424 operations were performed with 7 deaths. The anæsthetic table tells us that chloroform was given 200 times, nitrous oxide 116 times, A.C.E. mixture 92 times, ether 10 times, and cocaine 6 times.

